# Synthetic Secoisolariciresinol Diglucoside Attenuates Established Pain, Oxidative Stress and Neuroinflammation in a Rodent Model of Painful Radiculopathy

**DOI:** 10.3390/antiox9121209

**Published:** 2020-11-30

**Authors:** Sonia Kartha, Christine L. Weisshaar, Ralph A. Pietrofesa, Melpo Christofidou-Solomidou, Beth A. Winkelstein

**Affiliations:** 1 Department of Bioengineering, University of Pennsylvania, Philadelphia, PA 19104, USA; skartha@seas.upenn.edu (S.K.); christineweisshaar28@gmail.com (C.L.W.); 2 Department of Medicine, Perelman School of Medicine, University of Pennsylvania, Philadelphia, PA 19104, USA; ralphp@pennmedicine.upenn.edu (R.A.P.); melpo@pennmedicine.upenn.edu (M.C.-S.); 3 Department of Neurosurgery, Perelman School of Medicine, University of Pennsylvania, Philadelphia, PA 19104, USA

**Keywords:** analgesia, neuroinflammation, radiculopathy, secoisolariciresinol diglucoside, oxidative stress, pain, antioxidant, lignan

## Abstract

Painful cervical radiculopathy is characterized by chronic neuroinflammation that lowers endogenous antioxidant responses leading to the development of oxidative stress and pain after neural trauma. Therefore, antioxidants such as secoisolariciresinol diglucoside (SDG), that promote antioxidant signaling and reduce oxidative damage may also provide pain relief. This study investigated if repeated systemic administration of synthetic SDG after a painful root compression reduces the established pain, oxidative stress and spinal glial activation that are typically evident. SDG was administered on days 1–3 after compression and the extent of oxidative damage in the dorsal root ganglia (DRG) and spinal cord was measured at day 7 using the oxidative stress markers 8-hydroxguanosine (8-OHG) and nitrotyrosine. Spinal microglial and astrocytic activation were also separately evaluated at day 7 after compression. In addition to reducing pain, SDG treatment reduced both spinal 8-OHG and nitrotyrosine, as well as peripheral 8-OHG in the DRG. Moreover, SDG selectively reduced glial activation by decreasing the extent of astrocytic but not microglial activation. These findings suggest that synthetic SDG may attenuate existing radicular pain by suppressing the oxidative stress and astrocytic activation that develop after painful injury, possibly identifying it as a potent therapeutic for painful radiculopathies.

## 1. Introduction

Chronic neck pain remains a major concern for the aging population, with up to two-thirds of adults in the United States experiencing neck pain in their lifetime [1]. Radicular pain resulting from trauma to the spinal nerve roots is a common etiology of neck pain and can result from disc herniation, spinal stenosis, degeneration, and/or other disc trauma in the cervical spine. Animal models of radiculopathy from nerve root compression suggest that in addition to the local axonal injury and disruption in neurotransmitter release in the spinal cord [2,3,4], there is also widespread inflammation that contributes to the initiation and maintenance of chronic pain [5,6,7]. The initiation of robust inflammatory cascades both locally at the injury site and in the central nervous system (CNS) also leads to production of reactive oxygen species (ROS) and reactive nitrogen species (RNS) [8,9,10] that cause substantial cellular oxidative damage and further exacerbates the pathophysiology that potentiates radicular pain [11]. In addition to ROS accumulation, neural injury is accompanied by a decrease in the endogenous antioxidant enzymes that are activated in an effort to reduce cellular oxidative stress [9,11,12]. Although mounting evidence points to the contribution of oxidative and nitrosative stress in chronic radicular pain [11,13], few antioxidant therapies are effective in both addressing the generation of ROS and in reactivating endogenous antioxidant enzymes after neural injury.

Proinflammatory cytokine-induced generation of ROS [10,11] can activate several different intracellular stress pathways after painful neural injuries, including those that lead to further free radical formulation and DNA damage [8]. In a balanced reduction-oxidation (redox) state, the increase of free radical generation and ROS enhances the transcription of endogenous antioxidant enzymes [12]. Yet, loss of redox homeostasis with excessive ROS and RNS accumulation alters a cell’s ability to mount an effective antioxidant response and leads to a loss in regulation of cellular metabolism signaling [12,14,15]. Within the CNS, oxidative stress induces neuronal mitochondrial dysfunction that results in low ATP production leading to apoptosis and axonal degeneration [16]. Neurons are particularly susceptible to ROS and RNS species due to their high phospholipid content and their reliance on axonal mitochondrial transport for survival [10,13]. Chronic loss of redox homeostasis also leads to neuronal DNA damage, which has been increasingly linked to neurodegenerative diseases including Alzheimer’s and Parkinson’s disease [17,18], and more recently to radicular pain [17]. Expression of the DNA damage marker, 8-hydroxy-2-deoxyguanosine (8-OHG) increases in the dorsal root ganglia (DRG) after painful nerve root compression [17]. However, whether DNA damage occurs in the spinal neurons that are responsible for nociceptive processing [19] after painful radiculopathy is not known. Similarly, the contribution of nitrosative stress species like the potent oxidant, peroxynitrite, has been shown to be pro-nociceptive in several animal models of pain [8,20,21]. Although spinal nitrotyrosine, a downstream product and biomarker of peroxynitrite activity, increases in inflammatory pain [20], the spinal nitrotyrosine responses after painful radicular injury are not defined.

Accumulation of ROS and RNS, along with decreased transcription of endogenous antioxidant defenses, further promote a proinflammatory response in the CNS [22] leading to immune cell recruitment and cytokine release [8]. In fact, redox balance is essential for neuroinflammatory responses [14]. Given the role of inflammation and oxidative stress in chronic radicular pain [11,12,13,16,20,21,22], therapies that reduce oxidative stress and modulate the cellular redox state may be potent against the robust neuroinflammatory response that maintains chronic radicular pain [5,6,7,10]. Secoisolariciresinol diglucoside (SDG), a natural bioactive lignan from flaxseed, has been shown to be a potent free radical scavenger, antioxidant and anti-inflammatory agent [23]. Flaxseed lignan formulations enriched with SDG confer lung radioprotection [24] and mitigate lung damage when given after radiation exposure in mice [25,26,27]. Furthermore, synthetic formulations of SDG have comparable free radical scavenging and antioxidant properties to the natural, extracted SDG as confirmed by DNA radioprotective and radiation-mitigating properties in cell free systems [28] and in normal lung cells [29]. To date, preclincal testing of synthetic SDG has been performed in mice [30,31], non-human primates [32] and in an ex vivo lung organ culture model of proton-irradiated human lung [33], but not in painful radiculopathy. In addition to its free radical scavenging ability, synthetic SDG boosts endogenous antioxidant enzymes, such heme oxygenase-1 (HO-1), glutathione S-transferase µ1 (GSTM1) and NADPH:quinone oxidoreductase-1 (NQO1) [29]. Additionally, synthetic SDG has direct myeloperoxidase (MPO)-inhibiting activity [34]. MPO is highly-expressed in neutrophils, monocytes and some tissue macrophages [35,36], making up 2–5% of cellular proteins, and is responsible for the generation of the damaging hypochlorous acid (HOCl) associated with oxidation of macromolecules (lipids, proteins, DNA/RNA). Inhibition of MPO by synthetic SDG may be one of the mechanisms by which it inhibits inflammatory tissue damage.

Since SDG is capable of both detoxifying harmful oxidative stress species as well as inducing the endogenous antioxidant response in neural tissues, this study investigated the effects of SDG on modulating existing radicular behavioral sensitivity (i.e., pain), as well as its effects on the oxidative stress and inflammatory pathways that are activated when pain is present [10,13,17,20]. Using an established rat model of painful radiculopathy, SDG was administered systemically after a painful nerve root compression and the extent of neuronal 8-OHG labeling was evaluated by immunohistochemistry in the peripheral DRG and the spinal cord at day 7 after injury when pain and inflammation are typically evident [5,6,17,37]. In addition, spinal nitrotyrosine expression and activation of microglia and astrocytes were also evaluated at day 7 using immunohistochemistry in order to evaluate whether SDG treatment alters nitrosative damage and/or spinal glial activation.

## 2. Materials and Methods

### 2.1. Surgical Procedures and SDG Administration

All experimental procedures were approved by the University of Pennsylvania Institutional Animal Care and Use Committee (approved protocol number 805749) and carried out under the guidelines of the Committee for Research and Ethical Issues of the International Association for the Study of Pain [38]. Synthetic SDG (referred to as LGM2605) was generated as previously described [23]. Briefly, secoisolariciresinol diglucosides (*S*,*S*)-SDG (the major isomer in whole grain flaxseed) and (*R*,*R*)-SDG (the minor isomer in whole grain flaxseed) were synthesized from vanillin via secoisolariciresinol and glucosyl donor (perbenzoyl-protected trichloacetimidate under the influence of TMSOTf) through chromatographic separation of diastereomeric diglucoside derivatives (Chemveda Life Sciences, Inc, Hyderabad, India). Surgical procedures were performed using male Holtzman rats (275–299g; Envigo; Indianapolis, IN, USA). Rats received a painful nerve root compression (NRC; *n =* 13) or a sham surgical procedure (*n =* 6) as previously described [2,13,17,37]. Briefly, under inhalation isoflurane anesthesia (4% induction; 2–3% for maintenance), a midline incision was made over the cervical spine and a right dorsal hemilaminectomy at C6/C7 exposed the right C7 dorsal nerve root. A 10gf microvascular clip (World Precision Instruments; Sarasota, FL, USA) was inserted through a small opening in the dura to compress the nerve root for 15 min. After compression, the clip was removed, and the incision was closed using 3-0 polyester suture with surgical staples. Surgical sham control procedures included all of the same procedures except there was no root compression.

Rats were monitored after surgery and allowed to recover in room air while on a heating pad. Starting one day after surgery, rats were randomly assigned to receive synthetic SDG (200 mg/kg; subcutaneously repeatedly on days 1–3 in the afternoon after either a painful NRC (NRC+SDG; *n* = 8) or sham surgery (Sham+SDG; *n* = 6). For the remaining subset of the rats (*n* = 5) that received the painful NRC, a dose of phosphate buffered saline (PBS), which serves as the vehicle for reconstituting the synthetic SDG, was similarly administered subcutaneously on days 1–3 (NRC+PBS) to serve as vehicle controls.

### 2.2. Pharmacokinetic Evaluation of SDG in Rat Plasma

LGM2605 (at a dose of 100 mg/kg ) has been tested using multiple routes of systemic administration, such as intraperitoneal injection and oral gavage, and has been shown to significantly reduce inflammation and oxidative stress in murine models of septic cardiomyopathy [31] and neuroinflammation [30]. To evaluate SDG bioavailability in circulating plasma, synthetic SDG (200 mg/kg) was administered subcutaneously one day after a painful NRC (NRC+SDG; *n* = 3) and also to naïve rats (SDG; *n* = 3). SDG’s bioavailability was measured by circulating plasma levels of SDG at 0, 0.5, 1, 2, 4, 8, and 24 h after administration of SDG. Plasma levels of SDG were determined by an AB SCIEX 4000 triple quadrupole mass spectrometer (SCIEX, Framingham, MA, USA) as described previously [39,40]. Plasma samples with SDG levels below the limit of quantification were taken as the lower limit of quantification for the assay; 5ng/mL. Plasma SDG data were analyzed using two-way analysis of variance (ANOVA) to test for the main effects of time and exposure (naïve vs. painful NRC), along with the interaction between these variables, on plasma SDG levels with post-hoc Tukey test.

### 2.3. Behavioral Assessment

Behavioral sensitivity was measured in response to mechanical stimuli in the ipsilateral forepaw as previously described [13,17,41,42] before surgery (baseline; day 0) and daily after surgery until day 7. On days 1–3, behavioral testing was performed in the morning before administration of SDG or PBS. For each test session, the forepaw was stimulated using a series of von Frey filaments of increasing strengths ranging from 1.4 g to 26 g (Stoelting; Wood Dale, IL, USA); this was repeated for 3 rounds with at least 10 min of rest between each round. The lowest strength filament to evoke a response was recorded as the response threshold if the next higher filament also elicited a response. If the rat was unresponsive to all filaments, the maximum filament strength (26 g) was recorded as the response threshold. Thresholds for each testing session were averaged for each rat on each day and compared using a repeated measures ANOVA with post-hoc Tukey test over time and between groups.

### 2.4. Tissue Harvest, Immunohistochemistry and Analyses

On day 7 after behavioral assessment, rats were deeply anesthetized with sodium pentobarbital (65 mg/kg) and transcardially perfused with PBS and 4% paraformaldehyde [13,17,37,41]. The C7 spinal cord and DRGs were isolated and post-fixed overnight then stored in 30% sucrose for 6 days at 4 °C prior to cryosectioning. Samples were axially sectioned at a 14 µm thickness onto slides for immunohistochemical labeling.

To evaluate the extent of oxidative damage in the spinal cord, neuronal 8-OHG was assessed by co-labeling 8-OHG with the neuronal marker NeuN. Nitro-oxidative species were also separately assessed using the marker nitrotyrosine. In separate runs, tissue sections were blocked in 10% goat serum with 0.3% Triton-X 100 (Vector Labs; Burlingame, CA, USA) and then incubated overnight at 4 °C in a primary antibody solution of either mouse anti-8-OHG (1:200; Abcam; Cambridge, MA, USA), anti-NeuN and 555 conjugate (1:500; Millipore; Billerca, MA, USA) or mouse anti-nitrotyrosine (1:250; Abcam; Cambridge, MA, USA). The next day, tissue sections were incubated in secondary antibody solutions containing goat anti-mouse 488 (1:1000; Life Technologies; Carlsbad, CA, USA) at room temperature for 2 h. Samples labeled for 8-OHG and NeuN were only exposed to goat anti-mouse 488 secondary antibody.

In addition, microglial and astrocytic activation were also assessed in spinal cord tissue using the markers of ionized calcium-binding adaptor molecule I (Iba1) and glial fibrillary acidic protein (GFAP) to label microglia and astrocytes, respectively. Tissue sections were blocked for 2 h in 10% goat serum (Vector Labs; Burlingame, CA, USA) and incubated in primary antibody solutions containing rabbit anti-Iba1 (1:1000; Wako; Richmond, VA) and mouse anti-GFAP (1:500; Millipore; Billerca, MA, USA) overnight at 4 °C. The next day, sections were incubated in a secondary antibody solution containing goat anti-rabbit 568 (1:1000; Life Technologies; Carlsbad, CA, USA) and goat anti-mouse 488 (1:1000; Life Technologies; Carlsbad, CA, USA). For all analyses, spinal cord and DRG samples were also collected from normal un-operated rats (*n* = 2) in order to provide reference for expression levels in naive control tissues. Tissue sections with no primary antibody also were included in all labeling protocols and analyses as negative controls and to verify specificity of each antibody.

Tissue sections (3–6 images each) were imaged for the DRG and spinal cord at 20x using a digital camera and stereomicroscope with DP2-BSW software (Olympus; Center Valley, PA) for each label. To assess 8-OHG labeling in the DRG, images of the ipsilateral DRG were cropped (450 × 450 pixels) to include 10–15 randomly selected neurons for each image; each image underwent intensity analysis of the labeling [17]. The mean signal intensity of each neuron was determined manually by outlining each neuron in ImageJ and then averaged across the neurons sampled for each DRG. The intensity of 8-OHG in each DRG was compared between groups using a one-way ANOVA with Tukey’s post-hoc test.

Spinal cord images were cropped to include only the superficial dorsal horn (750 × 150 pixels) on the side of the injury and quantitative densitometry was performed using a custom MATLAB (MathWorks; Natick, MA, USA) script to measure the percent positive pixels for each label [17,23,41,43]. Thresholds for positive fluorescence for each label were based on the intensity ranges for positive pixels in normal naïve tissue and used for each image for each label. To quantify localization of spinal 8-OHG in neurons, the total number of pixels positive for 8-OHG and NeuN was separately quantified, and neuronal 8-OHG was determined by dividing the total number of positive pixels for both 8-OHG and NeuN by the total number of positive pixels for only 8-OHG [17]. Spinal nitrotyrosine, Iba1 and GFAP labeling were separately determined by quantifying the percent positive pixels for each in the spinal cord. Each marker was compared across groups using a one-way ANOVA with Tukey’s post-hoc test.

## 3. Results

### 3.1. Plasma SDG Levels Increased after Subcutaneous Administration

**Figure 1:**
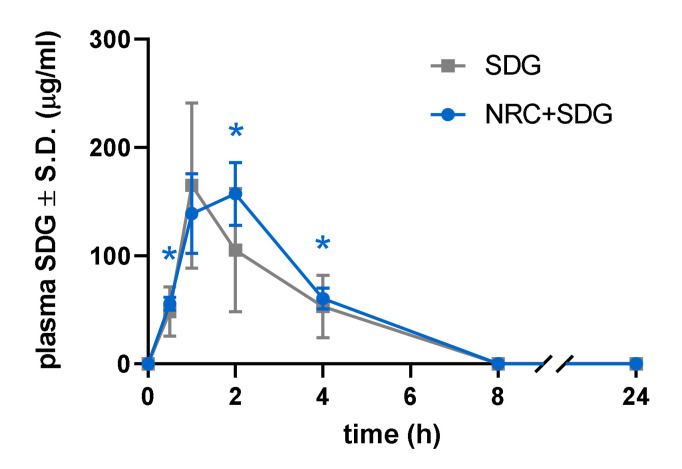
Plasma SDG levels significantly increase after a painful nerve root compression (NRC + SDG), as compared to levels at baseline (0 h, 0.5 h (* *p* = 0.0157), 2 h (* *p* = 0.0443) and 4 h (* *p* = 0.0329) after a subcutaneous injection. The levels do not differ from levels in naïve rats (SDG).

Plasma levels of intact SDG following a painful nerve root compression are significantly (*p* < 0.05) increased after subcutaneous dosing of 200 mg/kg SDG, peaking at 2 h post-injection (157.0 ± 29.1µg/mL) (Figure 1). Although plasma levels of SDG are still detected at 8 h and 24 h after drug administration, no significant differences are detected when compared to baseline. Overall, the kinetics of plasma levels of SDG do not differ between naïve rats and rats receiving a painful compression. Similar to previous pharmacokinetic findings with other routes of administration, the kinetics of SDG levels in plasma after subcutaneous drug dosing show levels peaking within 1–2 h after administration and returning to baseline by 8 h (Figure 1).

### 3.2. SDG Attenuates Established Behavioral Sensitivity

**Figure 2:**
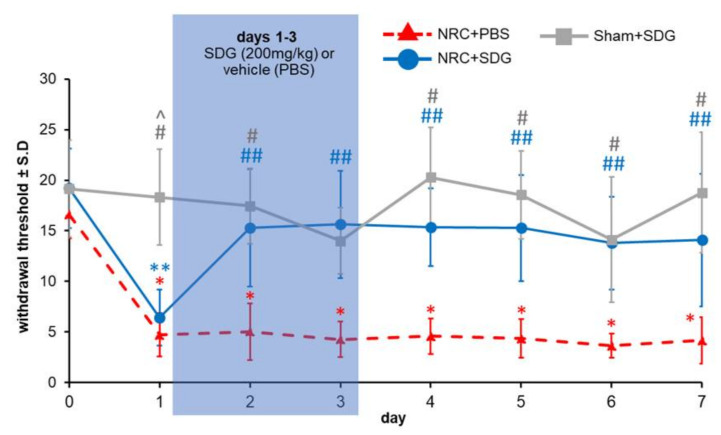
One day after a painful nerve root compression (NRC), withdrawal thresholds are significantly lower (i.e., more sensitive) than pre-injury baseline levels (day 0) regardless of whether they receive vehicle (* *p* < 0.0001) or SDG (** *p* < 0.0001) treatment. Withdrawal thresholds for the PBS vehicle group remain significantly lower (* *p* < 0.0001) than their baseline through day 7, while treatment with SDG significantly increases (^##^ *p* < 0.02) thresholds over vehicle as early as day 2, lasting until day 7. Thresholds after sham are significantly higher than both vehicle (^#^ *p* < 0.0004) and SDG(^^^ *p* < 0.0006) treatment at day 1 and significantly higher (# *p* < 0.016) than vehicle thresholds after day 1.

Repeated administration of SDG attenuates the behavioral sensitivity in the ipsilateral forepaw that typically occurs after a painful root compression (Figure 2). Immediately after a painful root compression, paw withdrawal thresholds are significantly lower (*p* < 0.0001) for both groups receiving that injury (NRC + SDG, NRC + PBS) compared to their baseline (day 0) (Figure 2). Yet, SDG treatment on day 1 significantly attenuates (*p* < 0.02) withdrawal thresholds compared to those of the vehicle (NRC + PBS) group within even one day, with increases in thresholds as early as day 2 and remaining elevated over through day 7 (Figure 2). The withdrawal thresholds in the treatment group are also greater than those of the Sham + SDG group, with no significant differences between groups on any day after SDG administration. Of note, withdrawal thresholds after sham procedures are not different from baseline at any time point and are significantly higher (*p* < 0.0006) than thresholds for both of the compression groups on day 1 and significantly higher (*p* < 0.016) than the vehicle group (NRC + PBS) on all days.

### 3.3. Oxidative Stress Markers in the DRG and Spinal Cord Decrease with SDG Treatment

**Figure 3:**
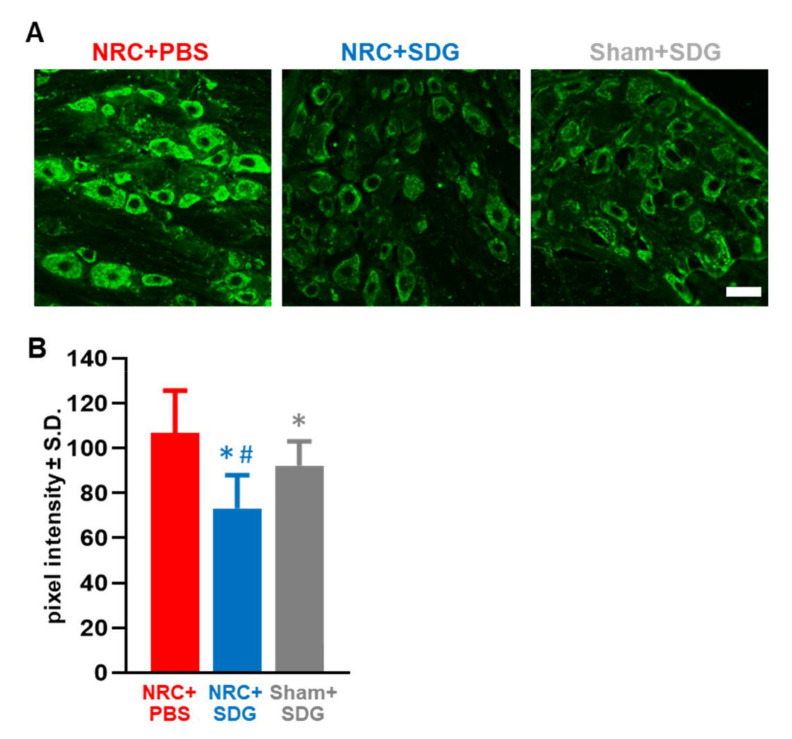
(**A**) Representative images show reduced 8-OHG labeling in the DRG with SDG treatment in rats receiving a painful NRC or sham surgery. The scale bar is 100 µm and applies to all panels. (**B**) After painful NRC, SDG treatment significantly decreases 8-OHG labeling compared to both NRC with PBS (vehicle) treatment (* *p* = 0.0001) and levels in the DRGs from Sham + SDG group (^#^ *p* = 0.0164). 8-OHG is also significantly lower (* *p* = 0.041) in the Sham+SDG treatment group compared to the NRC with vehicle treatment.

Paralleling the behavioral findings indicating SDG treatment returns thresholds to sham levels, repeated SDG treatment also reduces 8-OHG in DRG neurons at day 7 after a painful NRC to sham levels (Figure 3). In fact, the 8-OHG labeling with SDG treatment is significantly lower than expression in either the vehicle group (*p* = 0.0001) or the sham-treated group (*p* = 0.0164) (Figure 3B). Similarly, 8-OHG levels in the sham-treated rats are also significantly lower (*p* = 0.041) than corresponding levels in the vehicle NRC group.

**Figure 4:**
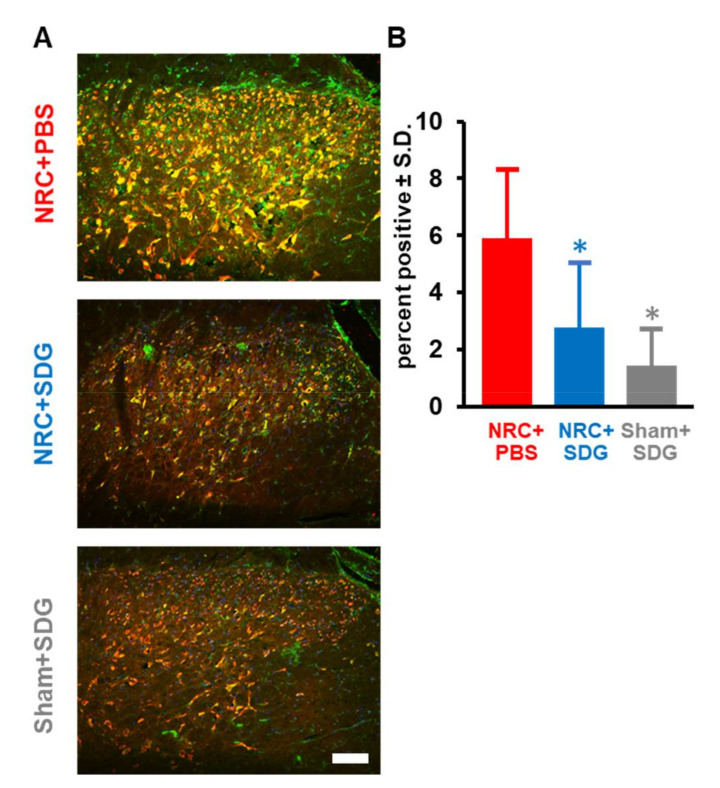
(**A**) Representative images show reduced neuronal 8-OHG labeling in the spinal cord in rats receiving either a painful NRC or sham surgery with SDG treatment (scale bar is 100 µm and applies to all panels). (**B**) Spinal neuronal 8-OHG labeling after a painful NRC is significantly reduced with SDG treatment (* *p* = 0.0001) to sham levels. Similarly, the sham SDG-treated group shows a significant reduction (* *p* = 0.0007) in spinal neuronal 8-OHG compared to the NRC + PBS group.

**Figure 5:**
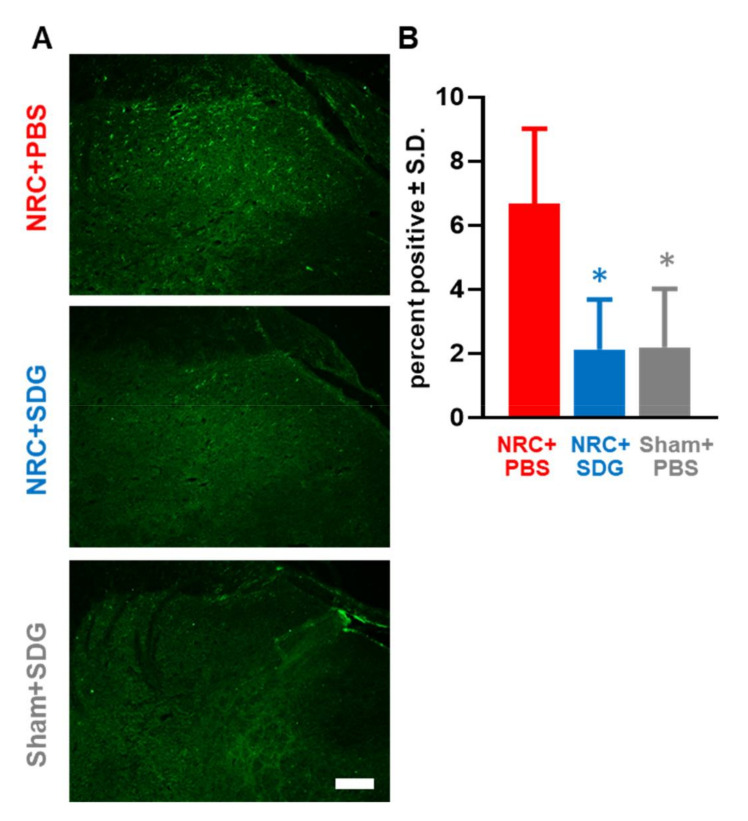
(**A**) After painful NRC, increased spinal nitrotyrosine expression is evident with PBS treatment but is lower after SDG treatment and similar to sham levels, as shown in representative images. The scale bar is 100 µm and applies to all panels. (**B**) Spinal nitrotyrosine levels after SDG treatment (*p = 0.0006) are significantly reduced to sham-treated levels. Spinal nitrotyrosine levels for a rat treated with SDG but undergoing a sham surgery are also significantly lower (* *p* = 0.0001) than the NRC+PBS group.

Spinal neuronal 8-OHG labeling after painful NRC is also reduced with SDG treatment (Figure 4A). Painful NRC increases spinal 8-OHG levels in the superficial dorsal horn by a 4.11 ± 1.87-fold from Sham+SDG levels (1.43 ± 1.29.0003) as observed in the NRC+PBS group (Figure 4A). Neuronal 8-OHG levels are significantly higher (*p* = 0.0007) after NRC than a sham surgery. However, treatment with SDG significantly lowers (*p* = 0.0001) neuronal 8-OHG labeling to sham-treated levels compared to the NRC group with PBS treatment (Figure 4). Likewise, SDG treatment also reduces the nitro-oxidative species, nitrotyrosine, in the spinal cord after painful injury (Figure 5A). Spinal nitrotyrosine expression increases by 2.87 ± 1.16-fold after a painful NRC over sham-treated nitrotyrosine expression; however, SDG significantly lowers (*p* = 0.0006) spinal nitrotyrosine to sham-treated levels (Figure 5B). Similarly, significantly less (*p* = 0.0001) spinal nitrotyrosine expression is observed in the sham group compared to the un-treated compression group.

### 3.4. SDG Reduces Spinal Glial Activation

**Figure 6:**
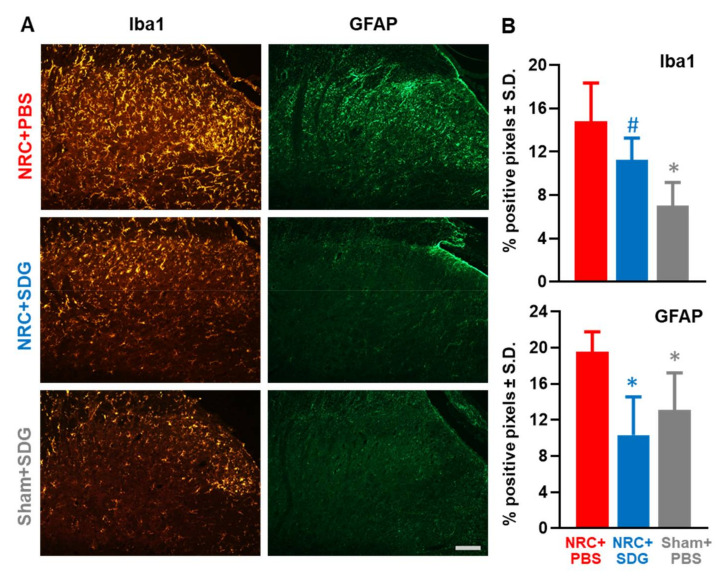
(**A**) SDG treatment after painful NRC reduces spinal astrocytic but not microglial activation, as evident in tissue sections from representative rats (scale is 100 µm and applies to all panels).(**B**) SDG treatment does not alter spinal Iba1 levels after NRC compared to vehicle treatment, with levels significantly elevated (^#^ *p* = 0.022) over sham-treated rats. Spinal Iba1 expression in the Sham + SDG group are significantly lower (* *p* = 0.0012) than the NRC+PBS group. After a painful NRC, spinal GFAP expression with SDG treatment is significantly reduced (* *p* = 0.003) compared to vehicle treatment. Similarly, spinal GFAP in sham-treated groups is significantly (* *p* = 0.03) lower than the vehicle treated group.

In addition to reducing oxidative stress responses in the spinal cord, SDG treatment modulates spinal glial activation to differing degrees (Figure 6). SDG treatment reduces the extent of spinal astrocytic, but not microglial, activation after painful NRC (Figure 6A.) Spinal Iba1 expression after SDG treatment is not different from expression in the NRC group receiving PBS vehicle and is significantly higher (*p* = 0.022) than expression in sham rats (Figure 6B). Spinal Iba1 levels are significantly lower (*p* = 0.0012) in the sham group than in the painful compression group with vehicle treatment. However, unlike its lack of effects on microglial activation patterns, SDG treatment does significantly reduce astrocytic expression (Figure 6). Spinal GFAP levels after SDG treatment are significantly reduced (*p* = 0.003) from levels in the vehicle group and are at sham-treated levels (Figure 6B). The extent of spinal GFAP expression in sham-treated rats is also significantly lower (*p* = 0.03) than expression in the vehicle treated compression group.

## 4. Discussion

This is the first study to demonstrate that synthetic SDG given early after the onset of radicular pain is sufficient to immediately abolish the behavioral sensitivity and peripheral and spinal oxidative damage that typically develop with such radiculopathy (Figure 2, Figure 3, Figure 4 and Figure 5). SDG treatment reduces 8-OHG labeling in neurons both in the DRG and spinal dorsal horn (Figure 3 and Figure 4), suggesting that neuronal oxidative damage may drive, at least partially, the peripheral and spinal cascades leading to chronic painful from nerve root trauma [2,6,13,17]. Further, since 8-OHG has been attributed to ROS-specific nuclear damage [44], the reduction in spinal nitrotyrosine (Figure 5) suggests that SDG may reduce oxidative damage from both ROS and RNS species. In parallel to reducing spinal oxidative damage, treatment with synthetic SDG decreases spinal GFAP-positive astrocytic activation (Figure 6B), suggesting it may preferentially modulate spinal neuroinflammation via astrocytic responses. Oxidative stress species, specifically ROS, initiate astrocytic activation in primary cultures astrocytes [45] and ROS-scavenging antioxidant therapies reduce spinal GFAP expression in chemotherapy-induced neuropathic pain [46]. Our findings establish systemically administered synthetic SDG as an effective antioxidant therapy for chronic radicular pain.

The finding that repeated systemic administration of SDG is effective in attenuating pain after nerve root compression (Figure 2) is consistent with the report that orally administered synthetic SDG provided pain relief from diabetic neuropathy in a mouse model [47]. However, in that study, SDG was only effective transiently, with behavioral sensitivity returning within three days of the cessation of SDG administration. While our study found no evidence of a transient response, it is not known how long the attenuation of radicular pain will last; longer time points are needed. Notably, in addition to the differences in etiology and pathophysiology between diabetic neuropathy and traumatic radiculopathy, the administration route for SDG also varied between the studies, which could have different pharmacokinetic effects. Nevertheless, synthetic SDG has been found to be more effective in reducing pain after peripheral nerve injury than comparable antioxidant therapies including ascorbic acid and α-tocopherol [48]. Even when administered repeatedly for three days and in combination after chronic constriction of the sciatic nerve, both ascorbic acid and α-tocopherol only produce an antinociceptive effect for two days. The greater analgesic effect of SDG may be explained by its approximate threefold greater reducing power than either ascorbic acid or α-tocopherol [23]. Taken together, SDG may better regulate oxidative stress and re-establish cellular homeostasis to attenuate radicular pain.

In conjunction with abolishing radicular pain, SDG reduces the peripheral and spinal oxidative damage caused by the ROS superoxide and RNS peroxynitrite that is evident after painful root compression (Figure 3, Figure 4 and Figure 5). Reductions in both neuronal 8-OHG and nitrotyrosine, which serve as surrogate markers for superoxide and peroxynitrite damage products [19,20,21], suggest SDG either directly or indirectly reduces accumulation of these putative pain molecules after neural injury. Dysregulation of superoxide and peroxynitrite are increasingly being considered as pro-nociceptive signaling molecules since they are implicated in the transition from acute to chronic pain in neuropathy [21]. The imbalance in redox activities and accumulation of superoxide and peroxynitrite in the peripheral and central nervous systems induces hyperexcitability in nociceptive neurons at the peripheral terminals of DRG neurons and central synapses in the spinal cord [49,50]. In the periphery, ROS enhance transient receptor potential (TRP) V1 activity in DRG neurons leading to enhanced firing of action potentials from nociceptive neurons [51]. In the spinal dorsal horn, both ROS and RNS can also directly disrupt glutamate homeostasis, including limiting the glutamate uptake in synapses and inhibiting its degradation through glutamine synthetase ultimately leading to excitotoxicity [52,53,54,55,56]. Together, these oxidative signaling in the periphery and spinal cord contribute to central sensitization, the increased sensitivity nociceptive neurons that maintain chronic pain states [5,46,57]. Since systemic administration of SDG reduces neuronal 8-OHG labeling in both the DRG and spinal cord (Figure 3 and Figure 4), may be an effective antioxidant treatment for pain relief by restoring the widespread oxidative and nitrosative balance.

Although antioxidant therapies focused on direct antioxidant compounds, and endogenous ROS scavengers like ascorbic acid have been tested in pre-clinical models of pain [39,58], those compounds have been found to have unfavorable pharmacokinetics. Newer antioxidant therapies have aimed not only to reduce oxidative damage but to also stimulate endogenous antioxidant responses [58]. Although SDG’s redox effect was not tested here, previous studies have confirmed SDG’s antioxidant potential in vitro [23,59,60,61]. In comparing natural and synthetic SDG with the known antioxidant alpha tocopherol (Vitamin E) with respect to their 2,2-diphenyl-1-picrylhydrazyl (DPPH) free radical scavenging activity, their free radical EC_50_ was found to be very similar [23,59]. SDG has also shown to decrease lipid peroxidation, and to decrease ROS, catalase and superoxide dismutase and glutathione peroxidase activity preventing heart hypertrophy and dysfunction in a rat model of pulmonary arteria hypertension which is known to have robust levels oxidative stress [62]. Synthetic SDG works via multiple pathways. In addition to directly scavenging ROS, specifically the hydroxyl radical, synthetic SDG increases nuclear factor erythroid-related factor 2 (Nrf2) signaling and induction of phase II antioxidant enzymes [29,59]. Upregulation of antioxidant, cytoprotective enzymes, such as HO-1 and NQO1, adds to the antioxidant capacity of cells to detoxify harmful ROS that are capable of damaging cellular DNA and other macromolecules. Nrf2 activation and boosting of endogenous antioxidant defenses are major mechanisms of action of nutritional antioxidants [63,64,65]. In conjunction with the ability of synthetic SDG to increase endogenous antioxidant enzymes, synthetic SDG is a potent inhibitor of the NLRP3 inflammasome, which generates the cytotoxic proinflammatory cytokine IL-1β upon activation, and whose pathway is one of the major inflammatory response pathways [66]. Synthetic SDG has also been shown to inhibit COX-2 as well as IL-1β and another potent inflammatory cytokine IL-6 in a human lung model of ischemia/reperfusion injury [67]. Importantly, synthetic SDG is also an inhibitor of nitrosative stress as shown by the reduction of total nitrates and the expression of inducible nitric oxide synthase (iNOS) in macrophages exposed to an inflammatory stimulus [68]. Although antioxidant enzyme expression was not explicitly tested here, assessing if systemic SDG administration increases endogenous cellular antioxidant responses in addition to reducing oxidative damage is needed to fully understand SDG’s effectiveness as a therapeutic after painful injury.

During states of redox homeostasis, the inflammatory response remains a repair and defense mechanism; yet, chronic oxidative stress alters intracellular cell signaling and dysregulates the neuroimmune response leading to the increased release of proinflammatory cytokines and downstream microglial and astrocytic activation [14,22] which exacerbate chronic neuroinflammation [69]. In addition to reducing both oxidative and nitrosative damage, repeated SDG treatment reduces at least one hallmark of neuroinflammation at day 7 (i.e., spinal astrocytic expression) (Figure 6). Previous studies have shown that flaxseed inhibits the release of pro-inflammatory cytokines [21,39], including TNF-α, which is known to activate both astrocytes and microglia and is upregulated within one day of this painful nerve root compression [70]. Therefore, as a potent ROS scavenger and inhibitor of cytokine release, SDG should reduce both astrocytic and microglial activation [4]. The observed elevation of Iba1 expression after SDG treatment (Figure 6) does not reflect or distinguish the microglial phenotypic changes that are tightly regulated by changes in ROS and RNS levels. The increased accumulation and production of oxidative stress species after injury lead to microglia adopting a pro-inflammatory M1 phenotype that is characterized by fewer, but thicker processes [69]. As microglial antioxidant responses increase, including increased transcription of Nrf2, microglial phenotypes shift from M1 to the anti-inflammatory, M2 phase [69]. Since Iba1 expression may not provide any information about phenotypic microglial changes, it remains unknown if SDG treatment reduces inflammation by promoting the shift of microglia to the M2 phenotype. More refined assessment of the different microglial populations after SDG treatment may provide more meaningful insights into the mechanisms by which SDG reduces inflammation, including enhancing the endogenous antioxidant response in addition to scavenging ROS.

Nevertheless, this study establishes SDG as a novel therapeutic to treat radicular pain, by detoxifying the accumulation of harmful oxidants as well as reducing the robust astrocytic activation that occurs post-trauma (Figure 3, Figure 4, Figure 5 and Figure 6). Most importantly, systemic treatment with SDG reduces both oxidative and nitrosative damage, suggesting that SDG may be a more effective antioxidant than other species-specific scavengers. SDG functions through free radical scavenging, as well as through multiple molecular pathways involved in endogenous antioxidant enzyme expression and regulation of the inflammatory response, while also being readily bioavailable and non-toxic. The antioxidant properties of SDG have been shown in cell free assays making it an effective “test-tube antioxidant” as discussed by Forman et al. [71] but have also been validated in other in vivo disease models. While it is noted that additional assays could be performed to more fully establish these effects in this painful injury model, SDG and its properties have been extensively reported elsewhere [23,24,25,26,27,28,29,30,31,32,33]. Although this study administered SDG early after injury, when the blood spinal cord barrier (BSCB) is known to be disrupted [72], SDG has been shown to readily cross the BSCB [73,74] and so is unhindered by invasive delivery challenges compared to other antioxidant therapeutics like fullerenes [75]. For these reasons and in the context of our current findings SDG may be a more effective antioxidant therapeutic for chronic radicular pain.
